# TiO_2_ Nanocomposite GelMA Film as Wound Dressing: Physicochemical, Structural, Mechanical Properties and Antibacterial Activity Against *Staphylococcus aureus*

**DOI:** 10.3390/nano16090536

**Published:** 2026-04-28

**Authors:** Barbara De Berardis, Raffaella Pecci, Roberta Morlino, Pietro Ioppolo, Marco Ranaldi, Giovanna Iucci, Alessandro Ferrarini, Giuseppe D’Avenio, Giorgio De Angelis, Maria Grazia Ammendolia

**Affiliations:** 1 National Centre Artificial Intelligence and Innovative Technologies for Health, Istituto Superiore di Sanità, 00161 Rome, Italy; raffaella.pecci@iss.it (R.P.); pietro.ioppolo@iss.it (P.I.); giuseppe.davenio@iss.it (G.D.); giorgio.deangelis@iss.it (G.D.A.); 2 Environment and Health Department, Istituto Superiore di Sanità, 00161 Rome, Italy; roberta.morlino@iss.it; 3 Department of Sciences, Roma Tre University, 00154 Rome, Italy; marco.ranaldi@uniroma3.it (M.R.); giovanna.iucci@uniroma3.it (G.I.); ale.ferrarini@stud.uniroma3.it (A.F.)

**Keywords:** GelMA, TiO_2_NPs, mechanical properties, structural characterization, drug release, antimicrobial activity, wound dressing

## Abstract

Bacterial infections can delay wound healing and represent serious medical problems both in the hospital and community settings, especially skin wound infections caused by *Staphylococcus aureus*. In this work, a gelatin hydrogel modified with photo-cross-linkable methacrylamide groups at 10% concentration (GelMA10%), enriched with titanium dioxide nanoparticles (TiO_2_NPs), and loaded with Neomycin sulphate was developed with the aim to realize a tissue for wound care with improved mechanical and antimicrobial properties. TiO_2_ nanocomposite GelMA films with two concentrations of TiO_2_NPs were characterized to assess physicochemical, structural and mechanical properties by scanning electron microscopy equipped with an energy-dispersive X-ray spectrometer (SEM/EDX), micro-computed tomography (micro-CT) and X-ray photoelectron spectroscopy (XPS). TiO_2_ nanocomposite GelMA films showed a more compact structure, reduced pore sizes and a higher compressive modulus at the increasing concentration of TiO_2_NPs. They were able to absorb and retain water for a prolonged time; however, no significant differences in the swelling degree at the increasing concentration of TiO_2_NPs were observed. *In vitro* drug release and antibacterial activity against *Staphylococcus aureus* of TiO_2_ nanocomposite GelMA film enriched with higher concentrations of TiO_2_NPs, identified as a suitable candidate for wound healing, were investigated. Both GelMA10% and TiO_2_ nanocomposite GelMA films loaded with drug exhibited a strong antibacterial action, whereas GelMA10% containing only TiO_2_NPs did not show any antimicrobial properties.

## 1. Introduction

Chronic wounds caused by burns, surgery and chronic diseases, such as diabetes, compromise the skin’s immune and protective mechanisms; as a result, bacteria can easily infect the wound [1]. Studies on microorganisms associated with wound infections showed that *Staphylococcus aureus,* followed by *Pseudomonas* spp. and *Escherichia coli* are the most prevalent pathogens on the wound surface [2].

*S. aureus* infections constitute a major public health concern, with approximately 30% of the population harboring the bacteria colonized in many bodily organs, including the skin [3]. In the clinical setting, methicillin-resistant *Staphylococcus aureus* (MRSA) is found in more than 40% of chronic wounds [4,5]. Chronic wounds, such as diabetic ulcers, arterial insufficiency ulcers, venous ulcers, and pressure ulcers, are wounds that do not heal for more than 6 weeks and are often caused by persistent bacterial infections and inadequate management. *S. aureus* infections can delay the healing process by producing a biofilm, resulting in antibiotic resistance and therapeutic complications [2], along with persistence due to intracellular colonization, which further promotes ongoing inflammation and continued activation of matrix metalloproteinases (MMPs) [6].

Traditional treatments of wound infections with antimicrobials and wound dressings made of cotton wool, film, and cotton gauze showed some limitations, such as inability to provide a moist environment around the wound and low resistance to bacterial infection [7]. Increasing demands have been made to develop non-cytotoxic wound dressings with intrinsic antimicrobial properties able to adsorb and remove the exudate, keep high moisture at the dressing–wound interface, allow gaseous exchange, and provide thermal insulation. For these reasons, research on the development of hydrogel matrices has gained great interest in the scientific community. Hydrogels, composed of hydrophilic polymer networks, mimic natural tissue environments, enhancing cell viability and function. In wound healing, hydrogels provide a moist environment, promote cell migration and, due to their porous structure, enable the diffusion and delivery of bioactive agents and antibiotics, fostering skin regeneration and repair [8].

Gelatin hydrogel matrices, modified with photo-cross-linkable methacrylamide groups (GelMA) to obtain photopolymerizable hydrogels with polymeric networks resembling that of an extracellular matrix, have been extensively investigated by the scientific community. The scientific interest is related to biocompatibility, biodegradability, stability, swelling capacity, and ability to maintain a moist wound environment of GelMA [9]. Moreover, the structural and swelling characteristics of hydrogel matrices make GelMA able to act as effective delivery vehicles for bioactive compounds, permitting the localized and sustained release of drugs. Neomycin sulphate is a broad-spectrum antibiotic, effective against infections due to certain Gram-positive bacteria, such as *Staphylococcus aureus* [10], and more efficient against most Gram-negative organisms such as *Proteus vulgaris*, *Escherichia coli*, *Aerobacter aerogenes*, *P. aeruginosa*, etc. It is widely used in cutaneous formulations [11]. Due to its solubility in water and minimal systemic absorption, Neomycin sulphate is incorporated into hydrogel for topical application [10,12].

However, low mechanical stiffness, toughness, and large pore sizes of GelMA, which can lead to a burst release of the encapsulated therapeutics, strongly limit its biomedical application [13,14]. To overcome these limitations, different nanomaterials, such as graphene oxide (GO) [15], gold [16], mesoporous silica nanoparticles [17], nanoclay [18] and carbon nanotubes (CNTs) [19], have been incorporated into different hydrogels.

Another nanomaterial used in wound healing is titanium dioxide NPs (TiO_2_NPs), due to their biocompatibility and antimicrobial properties [20]. They have been incorporated or *in situ* generated into different polymers, such as chitosan, polyurethane membrane, and gellan gum, showing that the enrichment with TiO_2_NPs not only led to an increased mechanical stiffness and thermal stability of the composite, but also to enhanced antibacterial properties [21,22,23,24].

In this study a TiO_2_ nanocomposite gelatin hydrogel loaded with Neomycin sulphate for treating skin infections caused by *S. aureus* was developed. The mechanical strength provided from TiO_2_NPs and the use of Neomycin sulphate allowed us to obtain a gelatin hydrogel with good structural characteristics, controlled drug release and improved antibacterial activity. We chose a narrow concentration range of TiO_2_NPs between 0.5 and 1 mg/mL to limit NP agglomeration in the hydrogel, which resulted in the inconsistency of the mechanical properties, creating weaker regions within the heterogeneous structure of the hydrogel and stress concentration points under pressure [25,26].

The first phase of this research was devoted to the preparation of GelMA films and TiO_2_ nanocomposite GelMA films with two concentrations of TiO_2_NPs. This study aimed to identify the optimal concentration of TiO_2_NPs that would allow for a GelMA10% film with improved mechanical and antimicrobial properties and controlled drug release. For this reason, physicochemical, structural and mechanical characterization of GelMA films and TiO_2_ nanocomposite GelMA films, as well as their swelling properties, was evaluated. From the characterization results, the authors identified the optimal configuration of the TiO_2_ nanocomposite GelMA film.

In the second phase of this study, the optimized TiO_2_ nanocomposite GelMA film, loaded with antibiotics, was investigated for cell viability on human keratinocyte cells (HaCaT cells) and for antimicrobial properties on *S. aureus*.

## 2. Materials and Methods

### 2.1. Materials

Titanium anatase nanoparticles with a primary size < 25 nm (TiO_2_NPs) and Neomycin sulphate were acquired from Sigma-Aldrich Company Ltd., Gillingham, Dorset, UK; lyophilized GelMA with a 50% degree of methacrylation (Photoel 50% DS) and lithium phenyl-2,4,6-trimethylbenzoylphosphinate (LAP) photoinitiator were purchased from CELLINK, Gothenburg, Sweden.

### 2.2. Physicochemical Characterization of TiO_2_NPs

TiO_2_NPs were characterized by Dynamic Light Scattering (DLS) (Zetasizer Ultra instrument, Malvern Instrument, Malvern, UK) and SEM/EDX (FE-SEM Quanta Inspect, FEI Company, Eindhoven, The Netherlands) equipped with EDAX TEAM^TM^ EDS software (v.4.6.2001.0293, AMETECH, Inc., Beijing, China) to determine their main physical–chemical characteristics: hydrodynamic diameter, primary size, shape, size distribution, agglomeration state, surface charge and chemical composition.

TiO_2_NPs were dispersed in Milli-Q water, and the suspension was sonicated with a probe sonicator (Vibra-Cell VCX 750, Sonics & Materials Inc., Newtown, CT, USA) at 20% amplitude, with a 6.5 mm probe diameter, under temperature-controlled conditions for 13 min to reduce agglomeration. DLS measurements were performed on 1 mL of TiO_2_NP suspension in Milli-Q deionized water. The temperature was set at 25 °C and two minutes were waited before starting the analysis to equilibrate the samples. Three determinations were performed on each sample. The number of readings for each determination and the duration of each measurement were automatically set by the instrument software (ZS Xplorer Software, v. 51.52.00, Malvern Instruments, UK). For particle size analysis, intensity distribution data were considered. For each sample, the mean value of the hydrodynamic diameter (Z-Average) and the polydispersity index (PDI) were determined, thus obtaining information on the stability and agglomeration state of the suspensions obtained. Zeta (ζ) potential measurements were also performed to determine the surface charge of TiO_2_NPs. The measurements were conducted in triplicate by taking 750 µL from the suspensions and using an automatic protocol of the Zetasizer Ultra instrument (Zeta Potential Software, v. 1.2.0.91, Malvern Instruments, UK).

To characterize TiO_2_NPs by SEM/EDX, 0.5 mL suspensions were transferred onto polycarbonate filters with a diameter of 47 mm and a porosity of 50 nm. Portions of the obtained sample filters were mounted on SEM sample holders and coated with a thin Au film by cathodic sputtering. Sample analysis was performed by choosing a beam voltage of 20 KV. For each sample, more than one hundred particles were analyzed, and their shape and size were determined. EDX spectra were also acquired from the analyzed particles to determine the elemental composition of the NPs and the presence of any impurities.

### 2.3. Preparation of TiO_2_ Nanocomposite GelMA Film

To obtain the GelMA-based hydrogel film, 500 mg of lyophilized PhotoGel 50% DS was used, adopting a protocol slightly modified as indicated by the manufacturer.

A PhotoInitiator (PI) solution was first prepared, containing 60 mg of LAP photoinitiator in 12 mL of Dulbecco’s Phosphate Buffer Solution (DPBS). The solution was first heated to 60 °C and kept in stirring, then filtered with a sterile 0.22 µm syringe filter and heated again to 60 °C to complete the dissolution. A total of 5 mL of PI solution was added to the lyophilized GelMA, previously brought to room temperature, to obtain polymeric films with a final GelMA concentration of 10% (GelMA10%). Starting from GelMA10%, two nanocomposite films enriched with TiO_2_NPs were made, one at a concentration of 0.5 mg/mL and the other at 1 mg/mL of NPs, indicated as GelMA10%_0.5TiO_2_NPs and GelMA10%_1TiO_2_NPs, respectively.

**Figure 1:**
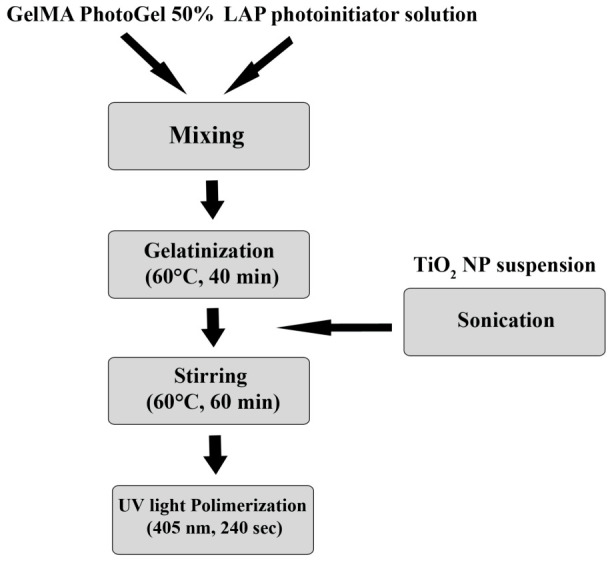
Schematic representation of GelMA and TiO_2_ nanocomposite film preparation.

Reconstituted GelMA with or without TiO_2_NPs was placed on a rotating plate at 60 °C for 1 h to promote complete dissolution. pH was then checked to ensure that it was between 7 and 7.4, balancing, if need, with small volumes of NaOH or HCl solutions. Two ml of each preparation was transferred into a cylindrical silicone mold, having a diameter of 2 cm, and photocrosslinked with a UV lamp at 405 nm and 4 cm distance for 240 s (Figure 1).

### 2.4. Characterization of GelMA and TiO_2_ Nanocomposite GelMA Film

After photo crosslinking, hydrogel films with a diameter of 2 cm and a thickness of 0.5 cm were obtained. We chose this thickness mainly for proof-of-concept testing. GelMA and TiO_2_ nanocomposite films were characterized from a physicochemical and mechanical point of view by SEM/EDX, XPS, micro-CT and mechanical compression tests to study their morphology, the distribution of TiO_2_NPs in the film, the film structure and the mechanical properties.

Moreover, the swelling behavior of GelMA and TiO_2_ nanocomposite GelMA films was investigated.

#### 2.4.1. Characterization by SEM/EDX Analysis

To better understand the role of TiO_2_NPs in the structure of GelMA-based films, cross-sections of freeze-dried samples of hydrogel films, with and without NPs, were characterized by SEM/EDX [13]. Films obtained after photo crosslinking were immersed for 24 h in DPBS to remove any non-crosslinked GelMA. After removing the DPBS, the samples were frozen in liquid nitrogen and freeze-dried for 24 h. The cross-sections of the freeze-dried samples were mounted on SEM stubs, coated with a thin Au film by sputtering, and analyzed by SEM at 20 KeV. Image editing was performed by Adobe Photoshop CS4 software (Adobe Systems, San Jose, CA, USA). Three areas of the samples were analyzed at a magnification of 1600×. More than 200 pores were measured.

#### 2.4.2. Characterization by XPS

Synchrotron radiation-induced X-ray photoelectron spectroscopy (SR-XPS) was employed to investigate the surface chemical composition and electronic structure of GelMA and Neomycin sulphate-loaded GelMA PhotoGel. SR-XPS experiments were performed at the TEMPO beamline (SOLEIL Synchrotron, Saint-Aubin, France) [27]. Spectra were collected in fixed analyzer transmission mode with a pass energy of 10 eV, utilizing a monochromator grating of 800 L/mm (energy range 330–825 eV). Core levels (C 1s, N 1s, O 1s, and S 2p) were acquired at a photon energy (PE) of 560 eV, with an overall energy resolution of ΔE = 0.1 eV. Samples were prepared in the solid state by drop-casting an EtOH/H_2_O (30:70 *v*/*v*) solution onto TiO_2_/Si (111) wafer surfaces. The energy scale was calibrated by referencing the C 1s aliphatic carbon signal to 285.0 eV [27]. Curve-fitting analysis was performed using Gaussian functions after the subtraction of a polynomial background [28,29]. To ensure physical consistency, a consistent fitting strategy, full width at half maximum (FWHM), was applied across all samples. For each core level, the same FWHM was imposed for all individual photoemission components. Calibration of the energy scale was made based on the C 1s core level signal of aliphatic carbons, found at 285.0 eV [27]. All peaks were imposed to have the same full width at half maximum (FWHM) within each core level to ensure physical comparability. For core levels subjected to splitting, constraints on the areas were imposed to respect the branching ratio, which is 2:1 for the S 2p_3/2,1/2_ core levels. S 2p spin-orbit splitting was imposed as 1.2 eV [28].

All atomic percentages are normalized to the total area of the corresponding element and therefore describe the relative distribution of chemical states, rather than absolute surface concentrations. Therefore, changes in relative percentages reflect chemical reorganization and screening effects, not simple additive behavior.

#### 2.4.3. Characterization by Micro-CT

Characterization of tissue scaffolds is important for tissue regeneration, and it is crucial to characterize both the mechanical properties and the internal microstructure of the scaffolds [30,31].

Micro-CT is commonly used as a 3D imaging method that provides sufficient depth and resolution for life science studies such as scaffold visualization [32,33,34]. Long scanning time is not suitable for studies involving soft tissues, since motion artifacts may be introduced into images due to the deformation during the scans. In addition to the aforementioned limitations, another critical challenge in conventional absorption-based micro-CT is that it is difficult to visualize low-density materials, such as hydrogel, due to the low X-ray absorption attenuation of these materials [35].

Therefore, it was decided to freeze-dry the samples so that they could be analyzed by microtomography and their internal structure could be studied. Morphological information, including internal structure, was obtained by micro-CT analysis performed on lyophilized samples using a high-resolution micro-CT scanner (Skyscan 1072; Bruker micro-CT, Kontich, Belgium). The resulting datasets (TIFF images) were acquired by TomoNT software (v.3N.5, Bruker micro-CT, Kontich, Belgium) using a resolution of 11.2 µm, with no filter, at a source voltage of 80 kV and a current intensity set at 124 µA, during a 180° sample rotation, with a rotation step of 0.45° and a frame exposure time of 1200 ms.

After the acquisition phase, the TIFF images were topographically reconstructed and analysed using dedicated software: NRecon (v. 1.7.0; Bruker micro-CT, Kontich, Belgium) and CT-Analyser (v. 1.16.9; Bruker micro-CT, Kontich, Belgium) respectively.

2D micro-CT images were processed by Adobe Photoshop CS4 software (Adobe Systems, San Jose, CA, USA) for editing.

#### 2.4.4. Mechanical Properties

To analyze the mechanical properties of the GelMA-based films and evaluate the influence of TiO_2_NPs, mechanical compression tests were performed on pure GelMA film and TiO_2_ nanocomposite GelMA films using a Lloyd Instruments LR 30K dynamometer (Lloyd Instruments Ltd., Part of Amptek STC, West Sussex, UK) at room temperature. Cylindrical samples with a diameter of 2 cm and a thickness of 0.5 cm were prepared for the compression test. GelMA-based films have been incubated in DPBS for 24 h and kept at 37 °C. Before mechanical testing, the samples were then blotted dry and placed between two parallel plates, having a diameter of 5 cm; the compressive force was applied parallel to the longitudinal axis of the samples with a speed of 1.0 mm/min.

The compressive modulus was calculated as the slope in the linear region of the stress–strain curve corresponding to 0–10% strain [36]. Two samples for each formulation of TiO_2_ nanocomposite films were tested, and for each sample, multiple measurements of compressive modulus were performed to calculate the average values.

#### 2.4.5. Swelling Kinetics

GelMA-based and nanocomposite GelMA films obtained by photo crosslinking with a UV lamp were weighed, obtaining the initial weight (*w*_0_) of the films after their fabrication [37]. Immediately after the fabrication, they were soaked in DPBS and stored in DPBS at 37 °C for 72 h. At intervals of 0.5, 1, 2, 4, 24, 48, and 72 h, they were removed from the DPBS, gently dried, and weighed, providing the weight (*w_t_*) at different time intervals [37]. From the difference in the weights obtained, we calculated the fluid absorption rate of each film using the following equation:

$$Fluid\;absorption\;rate\;\left(\%\right)=\frac{w_{t}-w_{0}}{w_{0}}\times 100$$

The degree of swelling was presented as a percentage of the maximum weight obtained for each film, independently of time, with respect to the initial weight *w*_0_ [37]. The assay was performed in triplicate, and the results were presented as the mean of experiments performed for each film.

### 2.5. GelMA and TiO_2_ Nanocomposite GelMA Film Loading

The objective of our study was to identify the optimal configuration of the TiO_2_ GelMA nanocomposite to obtain a hydrogel with improved mechanical properties while simultaneously exhibiting good swelling and ensuring controlled and sustained release over time by reducing the hydrogel’s pore size. To select the optimal configuration of the TiO_2_ nanocomposite GelMA film to be loaded with drug, experimental data relating to morphological/structural characterization, mechanical properties, and swelling were examined. Data on TiO_2_ nanocomposite GelMA films, obtained by SEM and micro-CT analysis, exhibiting a reduced porosity and smaller pore size and suggesting improved controlled drug release were selected. At the same time, the TiO_2_ nanocomposite GelMA showing the higher improvement in mechanical properties and the higher swelling degree was considered.

Neomycin sulphate was transferred in a GelMA-TiO_2_ reconstituted formulation containing PI solution and stirred for 20 min at 60 °C. Two ml of preparation were transferred into a cylindrical silicone mold and photocrosslinked, as described above, to obtain films containing 20 mg of Neomycin sulphate.

### 2.6. Drug Release of GelMA and TiO_2_ Nanocomposite GelMA Films

Standard curves of the drug were obtained by using the ultraviolet–visible spectrophotometer Lamda 35 (Perkin Elmer, Shelton, CT, USA). Neomycin sulphate was tested at 310 nm. The amount of Neomycin sulphate released was obtained from the standard curve.

The optimized GelMA and GelMA nanocomposite films were weighed and placed into 4 mL of DPBS buffer solution (pH = 7.4). The samples were stored at 37 °C, and at certain time intervals, 1 mL solution was taken out for UV-Vis measurements, and 1 mL of fresh DPBS solution was added to the samples [38]. The Neomycin sulphate release experiments were performed using GelMA films and TiO_2_ nanocomposite GelMA films without Neomycin sulphate loads as the blank controls to eliminate the possible influence from degraded hydrogel fragments and TiO_2_NPs. According to the data, the mass and percentage of drug release were obtained. The assay was performed in triplicate, and the results were presented as the mean of experiments performed for each film.

### 2.7. Cytotoxicity Test

To test the cytotoxicity of the nanocomposite films, an MTT assay was performed according to EN ISO 10993-5 [39], using HaCaT cells, a human skin cell line, obtained from ATCC (Manassas, VA, USA). Cells were seeded into 96-well plates and cultured in DMEM supplemented with 10% FBS for 24 h at 37 °C under a 5% CO_2_ atmosphere. The films were cut into 8 pieces (0.39 cm^2^ area, 0.5 cm thick), sterilized under UV light for 30 min, and each piece was added into DMEM supplemented with FBS to get extracts of the materials. The extraction was performed for 24 h at 37 °C. Cell culture medium was then removed, and extraction media obtained from the hydrogels at concentrations of 100, 50, 25 and 12.5% were added to the cell monolayers. After cells were cultured for 24 h, 100 µL of the MTT solution was added to each well after the removal of the extraction media, and the cells were further incubated in MTT solution for 3 h at 37 °C and 5% CO_2_. In viable cells, MTT can be reduced into formazan crystals inside the cells by mitochondrial dehydrogenase enzymes. The resulting formazan formed inside the cells was then dissolved in DMSO, and the blue–violet solution was quantified by measuring absorbance at 570 nm on a microplate reader (PerkinElmer, Boston, MA, USA). All materials’ extracts were tested for six averages, and data were collected from 3 independent experiments. Cell viability was expressed as a percentage (%) compared to the control untreated cells (0% extract treatment).

### 2.8. Antibacterial Activity

Antibacterial activity of GelMA films, unloaded or loaded with Neomycin sulphate, was determined by disk diffusion method and time-kill assay. *S. aureus* 6538p, a standard strain obtained from ATCC (Manassas, VA, USA), was used in this study. The bacterial strain was inoculated on tryptone soya agar (TSA, Oxoid, Basingstoke Hampshire, UK) and incubated at 37 °C for 24 h. Subcultures were kept in trypticase soy broth (TSB) supplemented with 15% glycerol and stored at −80 °C for later use.

#### 2.8.1. Disk Diffusion Test

The assay was conducted according to the Clinical and Laboratory Standards Institute (CLSI) guidelines [40] with slight modifications. A bacterial suspension with density corresponding to a McFarland 0.5 turbidity standard, which corresponds to 10^8^ CFU/mL, was swabbed onto TSA plates with a sterile cotton swab. A piece of each GelMA film, corresponding to 1/8 of the entire film disk (0.39 cm^2^ area, 0.5 cm thick), was applied on each TSA plate, together with a sterile filter paper disc of 6 mm loaded with 20 µL of Neomycin sulphate solution (final concentration 30 µg) as a positive control and a sterile filter disk loaded with TSB as a negative control. Each plate was then incubated for 24 h at 37 °C. Each determination was performed in triplicate. The diameter of the inhibition zone produced by the bacterial strain representing antibacterial activity was measured in millimeters.

#### 2.8.2. Time-Killing Growth Curve

A time-kill assay was performed with a macro-dilution procedure according to the method recommended by CLSI [40] with slight modifications. Bacterial cells were cultured overnight and diluted to approximately 10^5^ CFU/mL. A piece (0.39 cm^2^ area, 0.5 cm thick) of each GelMA film, unloaded or loaded with the antibiotic, was added into the inoculum suspension. Muller Hinton Broth (MHB, Merk Life Science U.R.L., Milano, Italy) inoculated with each bacterial strain without the test compound acted as the control in this experiment. The inoculum cultures were incubated at 37 °C. Samples (200 all) were removed from each inoculum culture at time points 0, 1, 2, 4, 8, and 24 h, and turbidity was recorded using a microplate reader (Perkin Elmer, Shelton, CT, USA) at a wavelength of 600 nm. At the same time intervals, sample aliquots from each culture were ten-fold serially diluted and subcultured on Muller Hinton Agar (MHA, Merk Life Science U.R.L., Milano, Italy) plates. Viable counts were calculated in the units of CFU/mL, and kill curves were plotted with time (h) against the logarithm of the viable count (log10 CFU). Each experiment was carried out in triplicate.

Statistical analysis was conducted using one-way ANOVA followed by Tukey’s post hoc pairwise tests (GraphPad Prism, Version 5.0). A *p*-value of less than 0.05 (* *p* < 0.05) was considered statistically significant.

## 3. Results and Discussion

### 3.1. TiO_2_NP Characterization

The hydrodynamic diameters, polydispersity index and Zeta potential of 1 mg/mL and 0.5 mg/mL TiO_2_NPs were determined. Results are displayed in Table 1.

**Figure 2:**
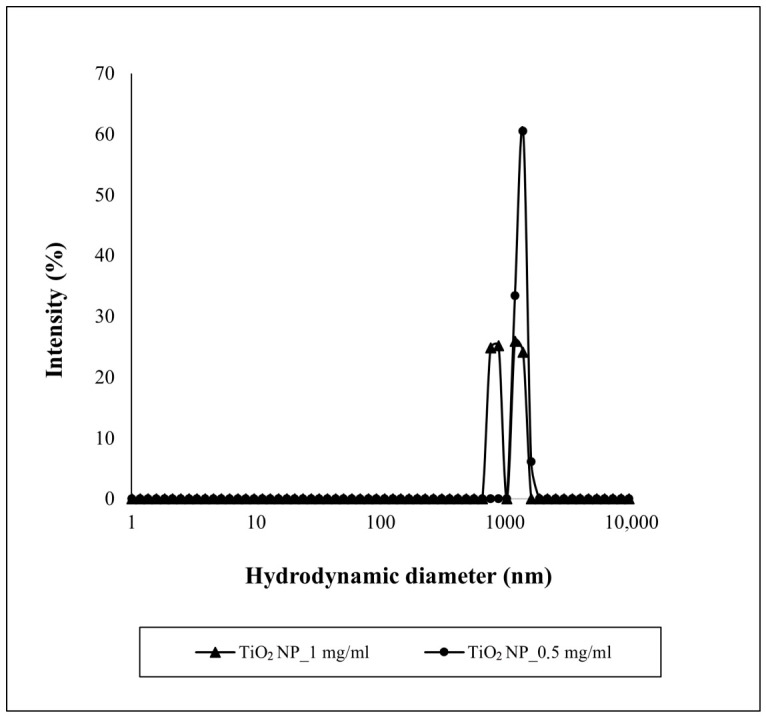
Size distributions by intensity of TiO_2_NP suspensions.

TiO_2_NPs showed an agglomeration state that increased with increasing suspension concentration, as indicated by high PDI values. Size distributions confirmed that, as indicated by PDI values. The size distribution of the TiO_2_NP suspensions at 0.5 mg/mL showed a maximum around 1.4 µm, and that at 1 mg/mL highlighted the presence of two peaks of practically equal intensity, one around 894 nm and the other around 1.2 µm (Figure 2). Moreover, TiO_2_NPs showed a negative surface charge (Table 1).

**Figure 3:**
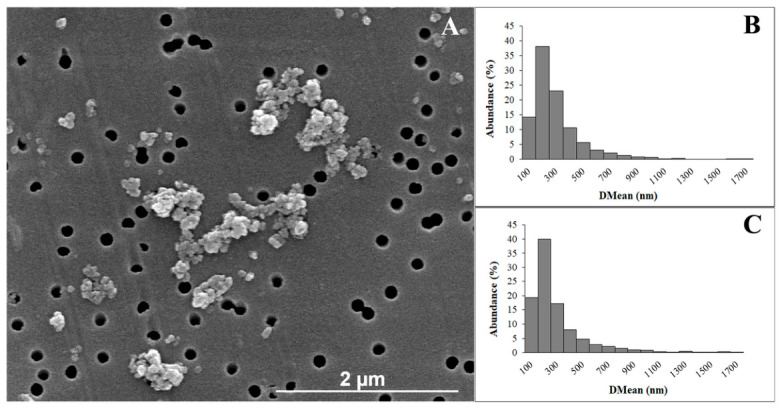
(**A**) Morphology of TiO_2_NPs; (**B**) size distribution of TiO_2_NPs at 0.5 mg/mL obtained by SEM analysis; (**C**) size distribution of TiO_2_NPs at 1 mg/mL obtained by SEM analysis.

SEM analysis displayed two main morphologies for TiO_2_NPs: a spherical one, with an average diameter between 20 and 60 nm, and an irregular one with a length of about 60 nm and a width of 40 nm. Size analysis by SEM confirmed what was suggested on the TiO_2_NP agglomeration state by the DLS data related to PDI. At the concentrations used in our study, the NPs formed agglomerates (Figure 3A) whose diameter was between 65 and 1700 nm. At 0.5 mg/mL, agglomerates smaller than 100 nm represent 19% of the analyzed NPs. A total of 65% of agglomerates had an average diameter between 100 and 400 nm (Figure 3B). At the concentration of 1 mg/mL, only 14% of the agglomerates have dimensions smaller than 100 nm and 72% between 100 and 400 nm (Figure 3C).

### 3.2. GelMA Film Characterization by SEM/EDX Analysis

**Figure 4:**
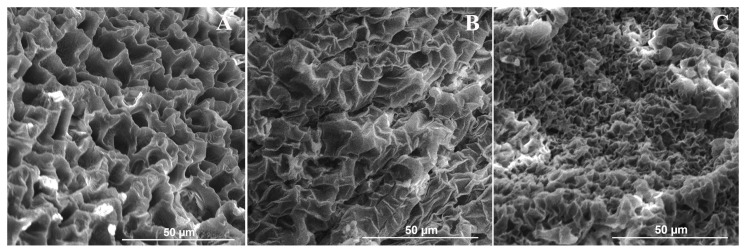
Morphology of GelMA and TiO_2_ nanocomposite GelMA film cross-section by SEM analysis: (**A**) GelMA10%; (**B**) GelMA10%_0.5TiO_2_NPs; (**C**) GelMA10%_1TiO_2_NPs.

SEM observations of lyophilized GelMA10% films showed an irregular and non-compact honeycomb structure with well-defined and largely non-interconnected pores with thin edges, indicating robust structural integrity (Figure 4A).

Several other studies observed this characteristic network of pores [13,41,42]. The mean diameter distribution of pores ranged from 8.6 to 41.6 µm, with an average value of 21.2 ± 9.0 µm. SEM observations of lyophilized TiO_2_ nanocomposite GelMA films showed the formation of parallel planes and a more robust and highly homogeneous network structure with increased pore wall thickness (Figure 3B,C). This uniform honeycomb structure plays a crucial role in facilitating the efficient transport and sustained release of encapsulated drugs [43]. Moreover, morphological analysis of lyophilized TiO_2_ nanocomposite GelMA films showed a dependence of pore sizes on TiO_2_NP concentration.

The mean diameter was between 9.1 and 36.6 µm with an average value of 20.7 ± 6.5 µm for GelMA10%_0.5TiO_2_NPs and between 7.0 and 24.4 µm with an average value of 13.5 ± 3.9 µm for GelMA10%_1TiO_2_NPs. Morphological features exhibited by lyophilized GelMA and TiO_2_ nanocomposite GelMA films indicated their ability to remove excess fluid and metabolic waste from the wound surface. Moreover, the decrease in pore sizes observed in the lyophilized TiO_2_ nanocomposite GelMA films suggest a higher crosslinking density with the increase in TiO_2_NP concentration and an increase in mechanical stiffness of the hydrogel [13].

**Figure 5:**
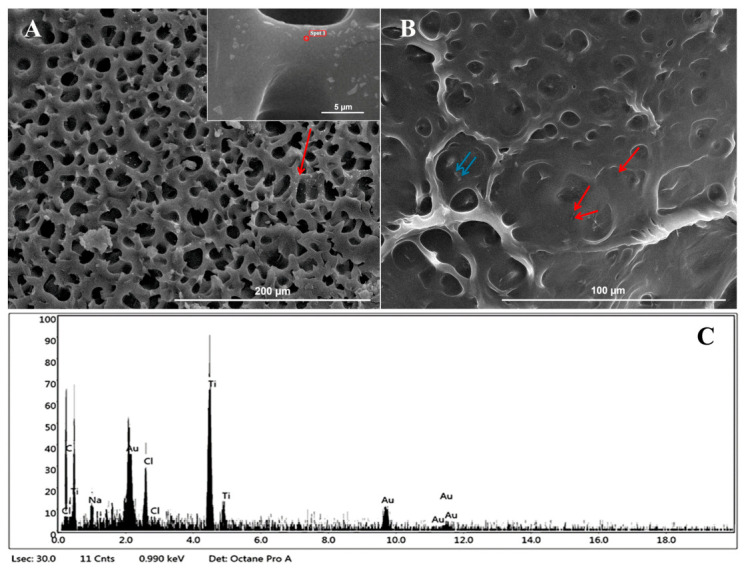
SEM/EDX analysis of TiO_2_ nanocomposite GelMA film: (**A**) SEM image of GelMA10%_1TiO_2_NPs’ cross-section; (**B**) SEM image of GelMA10%_1TiO_2_NPs’ surface; (**C**) typical EDX spectrum of TiO_2_NPs detected in GelMA. The arrows indicate small (red) and large (blue) agglomerates of TiO_2_NPs.

X-ray microanalysis spectra (Figure 5C) highlighted the presence of TiO_2_NP small agglomerates both in the cross-sections of the GelMA-TiO_2_NP nanocomposite films (Figure 5A) and on its surface (Figure 5B). Other studies observed hydrogel’s heterogeneous and rough surface due to the agglomeration of TiO_2_NPs [23,24].

### 3.3. XPS Analysis of GelMA and Neomycin Sulphate-Loaded TiO_2_ Nanocomposite Films

**Figure 6:**
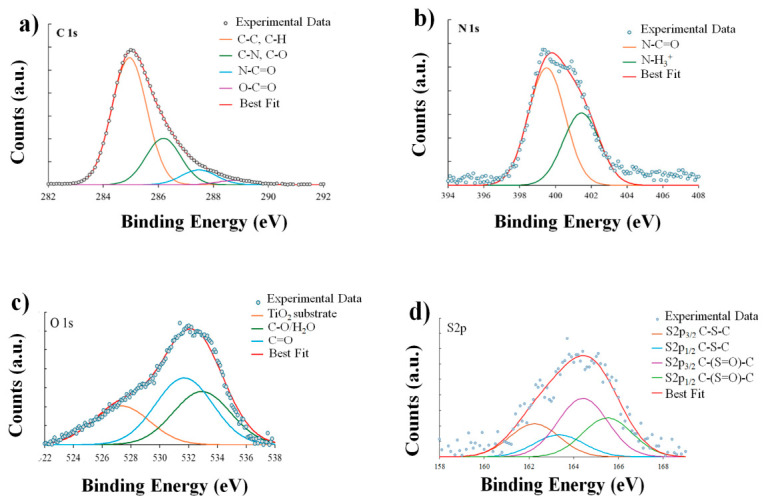
SR-XPS spectra collected on GelMA film: (**a**) C 1s, (**b**) N 1s, (**c**) O 1s and (**d**) S 2p core levels.

The chemical surface composition of GelMA films, deposited on TiO_2_/Si (111) from an EtOH/H_2_O (30:70) solution, was investigated via SR-XPS. This analysis served to characterize the pristine matrix, acting as a benchmark for monitoring spectral variations associated with the encapsulation of Neomycin sulphate. All measured spectra and spectral components individuated by applying a peak fitting procedure are shown in Figure 6.

All binding energy (BE), full width at half maximum (FWHM), atomic percentage values and proposed assignments are reported in Table S1 in Supplementary Materials. The C 1s spectrum is reported in Figure 6a. The envelope is broad and asymmetric, characteristic of the complex mix of amino acids and methacryloyl groups. The deconvolution identifies four distinct components: C-C at 285.0 eV BE, C-N and C-O at 286.18 eV BE, N-C=O at 287.5 eV BE and O-C=O at 289.1 eV BE.

The dominant component is located at 285.0 eV BE, assigned to aliphatic C–C and C–H (66%) bonds, originating from the hydrocarbon side chains of amino acids as well as the methyl and vinyl functionalities introduced by the methacryloyl modification. Moving to higher binding energies, the contribution attributed to C–N and C–O (24.1%) at 286.2 eV BE reflects the contributions of alpha-carbons (Cα) and peptide backbone linkages. The gelatine backbone is further characterized by the component at 287.5 eV BE, which corresponds to the amide carbonyls (N–C=O, 7.77%) of the peptide bonds connecting the amino acid sequence. Finally, the highest binding energy component at 289.5 eV BE is ascribed to carboxyl (O–C=O, 2.3%), resulting from the dual contribution of native carboxylic acid residues and the methacrylate ester linkages formed during the functionalization process. It is noteworthy that all oxidized C signals are also affected by surface impurities, always observed in samples prepared in air from water solution [28].

The N 1s spectrum (Figure 6b) shows two components: N-C=O at 399.5 eV BE and N-H_3_^+^ at 401.4 eV BE [44]. The predominant component, at 399.5 eV BE (64.2%), is ascribed to amide nitrogen (N-C=O) atoms; this signal arises directly from the peptide bonds along the gelatine backbone, confirming the structural integrity of the protein matrix. A secondary contribution is observed at 401.2 eV BE (35.9%), which is attributed to protonated amine species N-H3+. This latter signal is indicative of the presence of unreacted side-chain amines, such as those found in lysine or arginine residues, which exist in a charged state likely due to zwitterionic interactions or surface effects with the underlying substrate [44].

The O 1s spectrum (Figure 6c) analysis focuses on the organic layer, explicitly excluding the lattice oxide component detected at 529.6 eV, which arises from the underlying TiO_2_ substrate. Consequently, the organic oxygen contributions are resolved into two main components: the carbonyl oxygen (C=O) at 531.5 eV, associated with peptide amides and methacrylate esters, and the single-bonded oxygen (C–O) at 533.0 eV, attributed to hydroxylated residues.

Finally, Figure 6d shows the S 2p spectrum, which is characterized by the typical spin-orbit splitting doublet. The main S 2p3/2 component at 163.4 eV BE, the binding energy, is consistent with thioether linkages (C–S–C). This is typical of methionine residues within the gelatine backbone and indicates that the sulphur atoms remain in their native, non-oxidized form. Additionally, a secondary contribution is observed at 164.5 eV, which can be attributed to oxidized methionine species (such as methionine sulphoxide). This indicates that while a significant portion of the sulphur atoms remains in their native, non-oxidized form, a detectable fraction has undergone partial oxidation.

**Figure 7:**
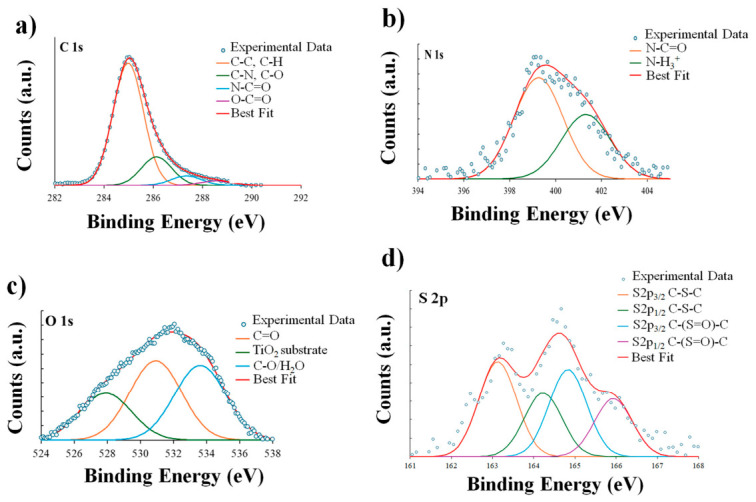
SR-XPS spectra collected on sample TiO_2_ nanocomposite GelMA films loaded with Neomycin sulphate: (**a**) C 1s, (**b**) N 1s, (**c**) O 1s and (**d**) S 2p core levels.

Following the characterization of the supporting matrix, the SR-XPS study focused on the nanocomposite GelMA loaded with Neomycin sulphate to verify the drug’s distribution and its chemical interplay with the polymer surface (Table S2 in Supplementary Materials). The C 1s spectrum for the Neomycin sulphate-loaded sample is reported in Figure 7a.

About the SR-XPS analysis of the Neomycin-loaded nanocomposite (Figure 7), the profile was deconvoluted into four chemically distinct environments, like the pristine matrix but with modified relative intensities. The C 1s spectrum (Figure 7a) reveals a significant increase in the aliphatic C–C/C–H component (74.3% vs. 66.0%), which reflects both the hydrocarbon contribution of the Neomycin structure and a relative screening of the protein backbone. This evidence is supported by the N 1s spectrum (Figure 7b), where the protonated amine fraction (N-H3+ at 401.3 eV) increases to 39.8%, serving as a diagnostic fingerprint of the drug’s amino groups and their electrostatic interplay with the matrix. Likewise, the O 1s spectrum (Figure 7c) shows an enhancement of the C–O signal at 533.6 eV, attributed to the aminoglycoside’s hydroxyl groups and retained water molecules. Finally, the S 2p data (Figure 7d) indicate that the methionine residues’ chemical identity is preserved; the persistent oxidized component at ~164.9 eV suggests that the loading process does not further alter the sulphur redox state relative to the pristine matrix. Although the overall profile remains like the pristine GelMA, a slight shift in the binding energies is observed.

Despite modulating the photon energy up to 1100 eV to increase the Inelastic Mean Free Path (IMFP) and enhance the sampling depth, no photoemission signal associated with the Ti 2p core level was detected. This suggests that the TiO_2_ concentration remains below the instrumental sensitivity threshold, likely due to a low surface density of the nanoparticles and the significant photoelectron attenuation (screening effect) exerted by the thick organic GelMA/Neomycin sulphate overlayer. The absence of a Ti 2p signal, even when using higher photon energy (1100 eV) to increase the Inelastic Mean Free Path (IMFP), provides crucial information regarding the nanocomposite’s architecture. Due to sampling depth < 10 nm, the absence of a Ti 2p signal indicates that the TiO_2_NPs were encapsulated by a thick organic overlayer of GelMA and Neomycin. Specifically, XPS analysis of the TiO_2_ nanocomposite GelMA film suggests that the outermost surface (the interface that interacts with the biological environment) is predominantly composed of the drug-loaded polymer matrix. The complete attenuation of the Ti 2p signal suggests a core-shell-like arrangement where the inorganic phase is deeply embedded, preventing its direct exposure at the surface. This observation is further supported by SEM/EDX analysis, which revealed a non-homogeneous distribution of the TiO_2_NPs within the film. Given the low overall concentration of TiO_2_NPs and their non-uniform spatial arrangement, the detection of the inorganic phase within the extremely shallow sampling depth of XPS (typically <10 nm) is reduced. Lastly, the XPS data proves that the addition of nanoparticles does not alter the chemical fingerprint of the Neomycin–GelMA interaction, as the observed shifts in N 1s and S 2p remain consistent with the drug-loading mechanism discussed in the text.

The XPS data confirm the successful loading of Neomycin sulphate within the GelMA matrix, as primarily demonstrated by an increase in the relative area of the C–O species within the O 1s spectrum compared to the pure GelMA baseline. Regarding the interaction mechanism, no significant binding energy shifts were observed for the N 1s or C 1s components, ruling out the formation of new covalent bonds. However, the retention of the drug within the substrate, coupled with the increase in the protonated amine fraction (N-H3+) is consistent with a physical encapsulation driven by electrostatic interactions between the polycationic aminoglycoside and the anionic hydrogel network [45].

Furthermore, the broad spectral envelopes observed for the O 1s and S 2p core levels reflect the high structural complexity and the inherently amorphous nature of the functionalized hydrogel network, consistent with established characterizations of gelatine-based biomaterials [10]. In particular, the extensive hydrogen-bonding network established between the multiple hydroxyl residues of Neomycin sulphate and the GelMA peptide backbone, along with the presence of residual bound water, induces a continuum of slightly shifted electronic environments. Such spectral characteristics underscore the chemical heterogeneity of the system, where methionine residues and oxygen-containing functional groups experience a variety of micro-environments dictated by the disordered arrangement of the methacrylated protein chains and the drug–polymer interface [46].

### 3.4. Micro-CT Analysis of GelMA and TiO_2_ Nanocomposite GelMA Films

**Figure 8:**
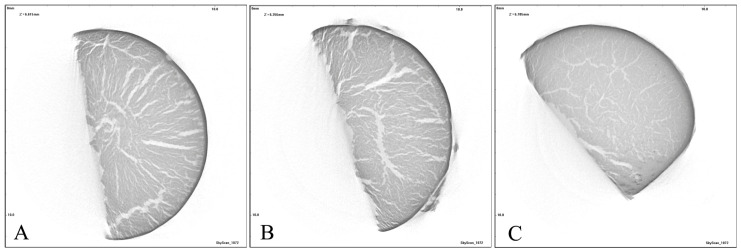
Micro-CT images of (**A**) GelMA10%; (**B**) GelMA10%_0.5TiO_2_NPs; and (**C**) GelMA10%_1TiO_2_NPs.

Starting from TIFF images obtained in the microtomographic acquisition step, the NRecon (v. 1.7.0) and CTAn (v. 1.16.9) software (Bruker micro-CT, Kontich, Belgium) were used to reconstruct the cross-sectional images and study the variation in the internal structure of the samples with varying amounts of TiO_2_NPs, as shown in Figure 8.

From the images, it can be observed that, by increasing the amount of TiO_2_NPs, the voids in the internal structure of GelMA10%_1TiO_2_NPs were significantly reduced (Figure 8C). A smaller amount of voids gives the sample greater compressive strength and therefore prevents rapid drug release. Subsequently, for each of the three freeze-dried samples, a dataset of a Volume Of Interest (VOI), constrained in terms of size, was extracted. In CTAn, the VOIs were analyzed through a procedure that included thresholding, noise reduction, and 3D analysis to determine and quantify the pore distribution within the three structures. The porosity values of GelMA10% and GelMA nanocomposites confirmed that with the increase in TiO_2_NP concentration, the structure of GelMA10% became more compact. In fact, the porosity of GelMA10% was 10.15%, while it was 12.26% and 5.11% for GelMA10%_0.5TiO_2_NPS and GelMA10%_1TiO_2_NPS, respectively.

### 3.5. Mechanical Properties of GelMA and GelMA Nanocomposite Films

The mechanical properties of GelMA-based films were regulated by TiO_2_NP concentration. Experimental data showed the compression modulus of GelMA10% reached the value of 4 kPa.

**Figure 9:**
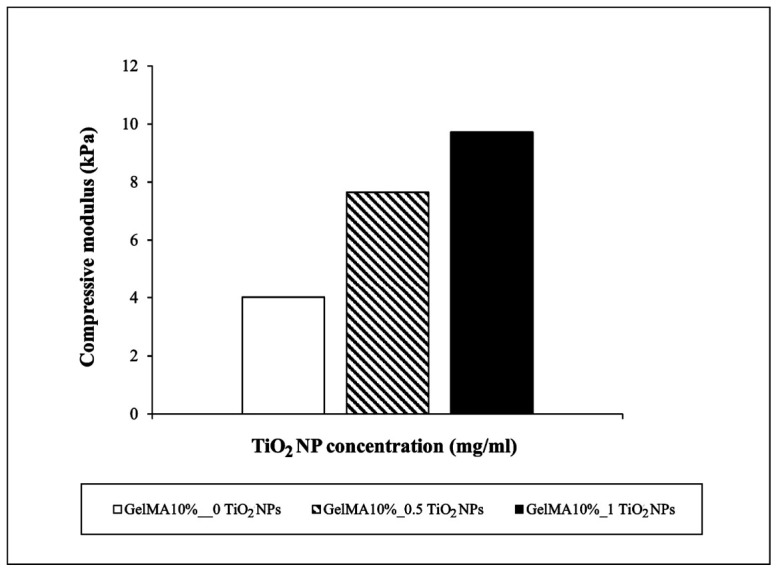
Effects of TiO_2_NP concentration on compressive modulus of GelMA10%.

The compressive stress–strain curves revealed that the presence of nanofiller led to significant improvements in the mechanical strength of the nanocomposite samples (Figure 9); the presence of TiO_2_NPs led to a significant increase in compressive strengths for all the nanocomposite samples compared to the GelMA10% sample.

Enrichment of 1 mg/mL TiO_2_NPs in GelMA10% led to a more than two-fold increase in the compressive modulus. Also, Ismail et al. (2019) [23] showed an improvement in compressive modulus for gellan gum biofilm incorporating TiO_2_NPs. The authors suggest that high surface interaction between TiO_2_NPs and biopolymer matrix leads to effective stress transfer between NP nanofiller and biopolymer chains.

Although literature data [25,26] indicate that the presence of agglomerates influences the consistency of the mechanical properties of hydrogels, our data on the compressive modulus of TiO_2_ nanocomposite GelMA films indicate that at the tested concentrations of TiO_2_NPs, the size of the agglomerates is such that it does not affect the consistency of the film proprieties.

### 3.6. Swelling Behavior

**Figure 10:**
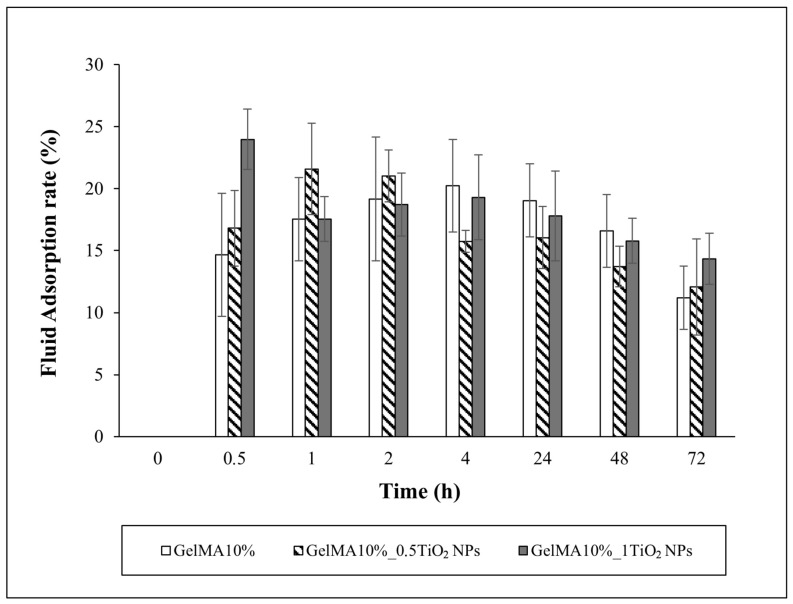
Swelling behavior of GelMA and nanocomposite GelMA films.

Swelling behavior of the GelMA film and TiO_2_ nanocomposite GelMA films are displayed in Figure 10.

GelMA10% films swelled up to 4 h, then the fluid absorption rate started to slowly decrease, probably due to polymeric erosion. The swelling of the films can be due to the hydrophilic nature of GelMA and the surface saturation that leads to the migration of water molecules into the film matrix, causing the chain expansion because of the weakening of the intramolecular hydrogen bonds [47]. The swelling degree of GelMA10% was 20.2% ± 3.7%. The addition of 0.5 mg/mL TiO_2_NPs led to an increase in fluid absorption rate up to 2 h, while a burst increase after 0.5 h was observed for GelMA10% enriched with 1 mg/mL of TiO_2_NPs. At 72 h, GelMA10%_1TiO_2_NPs exhibited a fluid adsorption higher than GelMA10%. No significant differences in the GelMA10% film enriched with TiO_2_NPs at the increasing of NP concentration were observed (Figure 9).

The swelling degrees of 21.6 ± 3.8% and 23.98 ± 2.4% for GelMA10%_0.5TiO_2_NPs and GelMA10%_1TiO_2_NPs, respectively, indicated a slight increase in the swelling behavior of GelMA enriched with TiO_2_NPs due to the hydrophilic nature of the anatase TiO_2_NPs.

Conflicting data on the swelling behavior of hydrogels incorporating TiO_2_NPs are available in the literature. Ren et al. (2015) [48] showed an increased water-binding capacity of PVA/xylan hydrogel enriched with TiO_2_NPs. Higher swelling was also observed in gellan gum film incorporating TiO_2_NPs. Authors suggested that the enrichment of TiO_2_NPs contributed to the higher surface area/volume ratio of the film, facilitating the adsorption of water and TiO_2_ molecules through the film [17]. In contrast, Gohargani et al. (2020) [49] demonstrated a significant decrease in swelling degree in biodegradable chitosan–whey protein-based film containing TiO_2_NPs.

Swelling behavior is a fundamental aspect of the wound healing of hydrogel films, since it determines the adhesion of the film to the wound, the ability to absorb exudates, and finally, the prolonged release of the encapsulated therapeutic treatments. On the whole, data indicated that GelMA10%_1TiO_2_NP films were suitable candidates for wound healing due to their ability to absorb and retain water for a prolonged time.

### 3.7. Neomycin Sulphate Release

A calibration curve was obtained using antibiotic solutions at 370 µg/mL, 750 µg/mL, 1250 µg/mL, and 2500 µg/mL. The UV spectra of Neomycin sulphate showed a peak at 310 nm.

**Figure 11:**
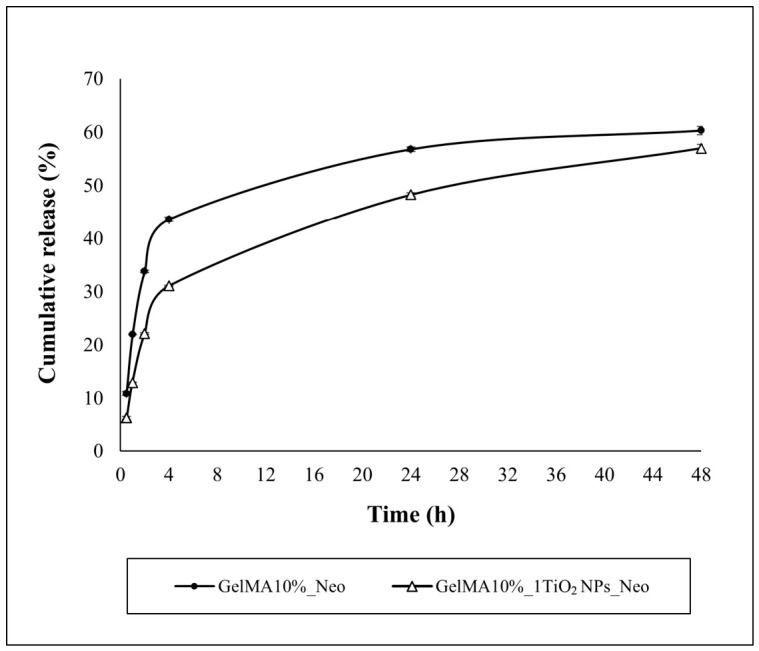
Cumulative Neomycin sulphate release of GelMA and TiO_2_ nanocomposite GelMA films.

The calibration curves of the antibiotic were y = 1.9549 × 10^−5^ x − 0.0074006, R = 0.99731. From the calibration curves, we estimated the cumulative Neomycin sulphate release (Figure 11).

The process of Neomycin sulphate release showed three stages in GelMA10% films. A burst release of drug was observed in the first 4 h, in which it could exert antibacterial action to protect the wound from secondary infection, followed by a slower release between 4 and 24 h, at which point about 57% of the antibiotic was released. After 24 h the slope of the cumulative drug release curve further decreased, and about 68% of Neomycin sulphate was released. Three stages were observed also in the process of release for GelMA10% film enriched with 1 mg/mL of TiO_2_NPs, but in each of three stages the release was slower than in GelMA10%. In the first stage (0–4 h), the slope of the cumulative release curve for the TiO_2_ nanocomposite GelMA film was lower than that observed for GelMA10%, whereas in the second, it was higher. This result agrees with the swelling behavior of GelMA10%, which swelled up to 4 h. The enrichment of 1 mg/mL TiO_2_NPs did not significantly affect the swelling properties of the GelMA film, but the observed reduction in pore sizes led to reduced and slowed diffusion of Neomycin sulphate and increased entrapment of the drug within the hydrogel meshes, thus inducing a more controlled drug release. Overall, these data indicate that the addition of TiO_2_NPs at a concentration of 1 mg/mL allowed us to obtain a controlled, sustained and prolonged release of Neomycin sulphate.

To understand the release mechanism, the cumulative release profile of GelMA10% and TiO_2_ nanocomposite GelMA film enriched with 1 mg/mL of TiO_2_NPs was fitted to the Ritger–Peppas model:

$$\frac{M_{t}}{M}=\;kt^{n}$$

where *M_t_* is the mass of the drug released at time *t*, *M* is the total mass of the drug, *k* is the kinetic constant, and n is the diffusional exponent. The *k* constant gives an indication of the burst release of the drug, while the diffusional exponent *n*, which gives information on the release mechanism, depends on the type of hydrogel geometry and polymer polydispersity [50].

For the GelMA10% film, we obtained k = 18.01 h^−1^ and n = 0.38, with R^2^ = 0.915. The diffusional exponent value obtained for our cylindrical geometry of the hydrogel matrix suggests a pseudo-Fick diffusion mechanism for GelMA10%, with a slow drug release [10]. While GelMA10% is highly porous, it acts with a pseudo Fick mechanism in which the relaxation of the GelMA polymer chains is very slow compared to the solvent diffusion time, reducing the initial burst effect to <30% in the first two hours, followed by a sustained release. This behavior is likely due to the polymer concentration, which has led to a relatively dense structure and a small free volume, and to the degree of cross-linking achieved, which makes the polymer chains difficult to separate. Also, Vigata et al. (2020) [51] observed a controlled release with a pseudo-Fick mechanism for GelMA10% concentration loaded with different doses of Abraxane. The authors showed that GelMA10% significantly reduced the amounts of released drugs, increasing the sustainability of the release, and the fraction of Abraxane released for all doses was not statistically different.

For GelMA10% enriched with 1 mg/mL of TiO_2_NPs, we obtained k = 11.86 h^−1^ and n = 0.46 with R^2^ = 0.931, indicating a lower burst release of Neomycin sulphate in the GelMA nanocomposite. The diffusional exponent values for the geometry of the hydrogel matrix suggest an anomalous transport phenomenon with comparable velocity of polymer chain relaxation and solvent diffusion, controlled by drug diffusion and erosion or relaxation of polymer chains [10]. This behavior is probably due to higher crosslinking density achieved by the enrichment of hydrogel with TiO_2_NPs, which led to a reduced pore size of GelMA10% and further slowed down the diffusion-driven release.

### 3.8. Biocompatibility of GelMA and GelMA Nanocomposite Films

**Figure 12:**
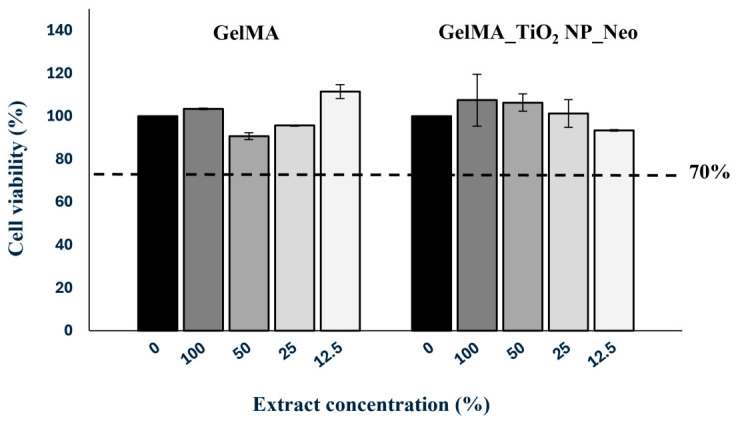
Cytotoxicity evaluation of hydrogel dressings on HaCaT cell monolayers using an MTT-based extract test.

According to the UNI EN ISO guidelines, the extracts eluted from the hydrogel dressings were used as the original extract at 100% concentration and as dilutions of the original extract at 50%, 25%, and 12.5% (*v*/*v*). Cytotoxicity test results showed that the viability of cells treated with extracts at all concentrations was greater than 70% (Figure 12, dotted line), which represents the cut-off suggested by the guidelines for the absence of a cytotoxic effect. The tested hydrogels therefore have non-cytotoxic potential and can be considered cytocompatible.

### 3.9. Antibacterial Activity of Optimized Nanocomposite GelMA Films

To evaluate the antibacterial action of GelMA nanocomposites loaded with Neomycin sulphate and possible synergistic effects with TiO_2_NPs, we evaluated optimized GelMA samples as the following scheme: GelMA10% film and GelMa10% film loaded with Neomycin sulphate, GelMA10%_1TiO_2_NP film and GelMA10%_1TiO_2_NP film loaded with Neomycin sulphate.

Disk diffusion test results indicated that GelMA and TiO_2_ nanocomposite GelMA films loaded with Neomycin sulphate significantly inhibited the growth of *S. aureus* compared to the blank control and GelMA films without antibiotics (Table 2). A comparable zone of inhibition for both GelMAs, with or without TiO_2_NPs, loaded with the antibiotic was evidenced, suggesting that the antibacterial activity was entirely due to the action of Neomycin sulphate, regardless of the TiO_2_NPs embedded into the gel dressing.

**Figure 13:**
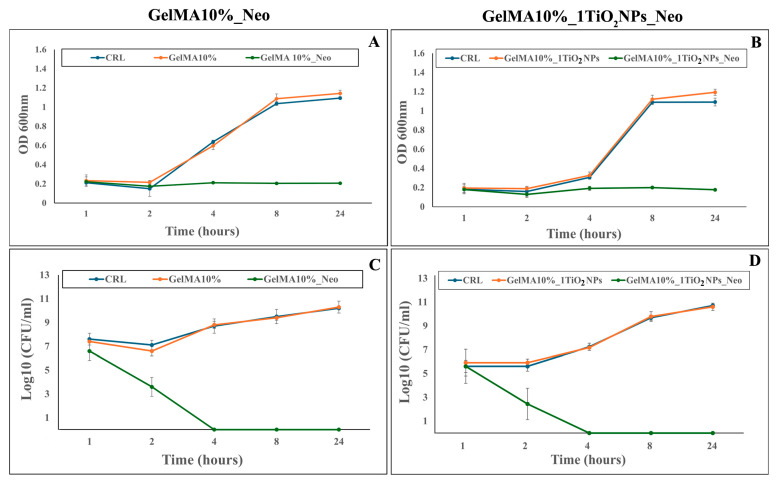
Effect of hydrogel specimens on the growth of the *Staphylococcus aureus* 6538p strain under exposure to GelMA10% (**A**,**C**) and GelMA10%_1TiO_2_NP (**B**,**D**) hydrogels, both unloaded and loaded with Neomycin sulphate.

Optical density curves confirmed the action of the antibiotic loaded into GelMA and GelMA_1TiO_2_NP nanocomposites, with bacterial inhibition starting from 1 to the 24 h time interval (Figure 13A,B).

Time-kill assay evidenced that the Neomycin sulphate released during the first 4 h of bacterial growth was able to reduce bacterial population by about 6 log10, compared to the control untreated sample and GelMA nanocomposites without Neomycin sulphate cultures (Figure 13C,D). GelMA nanocomposite made with TiO_2_NPs also in this assay showed antibacterial activity comparable to that of the GelMA nanocomposite without the nanoparticles, further confirming the absence of an antibacterial effect of TiO_2_NPs. In contrast with our data, other studies [23,24] observed antibacterial activity against both *S. aureus* and *E. coli* for gellan gum hydrogel incorporating green-synthesized TiO_2_NPs. The lack of antimicrobial activity of GelMA incorporating only TiO_2_NPs observed in our study is likely due to the low content of NPs loaded in the hydrogel (2 mg in the entire film) compared to TiO_2_NP concentrations used by Ismail et al. (2019) and Su et al. (2025) [23,24]. Moreover, the film integrity displayed for 72 h, consistent with the high crosslinking density and low relaxation of the polymer chains of GelMA10%_1TiO_2_NP, could prevent the NP release and subsequent antibacterial action. In addition, although the photo-cross-linking and film sterilization steps involving UV light should photoactivate the TiO_2_NPs with the production of free radicals, these are likely not sufficient to exert an antibacterial action in our model.

## 4. Conclusions

In this study, we developed a TiO_2_ nanocomposite GelMA film as a proof-of-concept to realize a wound dressing with a controlled and prolonged drug release and improved mechanical and antibacterial properties.

There are two studies available in the literature on TiO_2_ nanocomposite hydrogel based on GelMA [42,52]. Yang et al. [42] developed a 3D-printed antibacterial wound dressing incorporating N-halamine TiO_2_NPs in GelMA at 15% concentration and different concentrations of Xanthan gum to improve printing fidelity. Si et al. [52] developed a polymer/TiO_2_NP nanocomposite film in which TiO_2_NPs, at a weight percentage ranging between 0.5- and 4, were *in situ* generated in gelatin with a 79% degree of substitution, using Irgacure 2959 as a photoinitiator and a time of photocrosslinking of 30 min. In all these studies, TiO_2_NPs were used to provide antibacterial activity of GelMA.

The novelty of our study consists of some critical factors that affect the mechanical properties of hydrogel and cell viability, such as GelMA concentration, degree of substitution, photoiniziator and concentration of TiO_2_NPs. We used GelMA at 10% concentration, indicated as an optimum hydrogel concentration for various cell studies and bioprinting [53], and LAP as a photoinitiator that allows obtaining smaller pore sizes and slower degradation rates when compared to Irgacure 2959 [53]. Finally, to obtain a controlled drug release, we used a lower range of concentrations for TiO_2_Ns, between 0.5 and 1 weight percentage (0.5–1 mg/mL), to limit the agglomeration observed in other studies that may affect the mechanical properties of the hydrogel [25,52]. We loaded the optimized TiO_2_ nanocomposite GelMA film with Neomycin sulphate to provide antibacterial activity to the hydrogel, and we assessed the potential synergistic effect of TiO_2_NPs on the antibacterial activity of the film.

Our results showed that the enrichment of GelMA with 1 mg/mL of TiO_2_NPs (1 weight percentage) led to several advantageous properties: a compact and porous structure with smaller porosity and pore sizes, higher photo-cross-linking density and improved mechanical performance compared to GelMA10% hydrogel. The enrichment of GelMA10% with this low concentration of NPs made the TiO_2_ nanocomposite GelMA film a suitable candidate for wound healing due to its enhanced structural stability and ability to absorb and retain water for a prolonged time as indicated by swelling data. Finally, the incorporation of 1 mg/mL TiO_2_NPs in GelMA10% loaded with Neomycin sulphate provided a controlled drug release profile, reducing burst release and extending the duration of drug availability. From the collective results, it can be known that this optimized TiO_2_ nanocomposite GelMA film loaded with drug showed an excellent antibacterial ability against *S. aureus*. The amount of Neomycin sulphate released by GelMA10%_1TiO_2_NPs in the first 4 h was sufficient to inhibit almost all bacteria and ensure prolonged inhibition, as is desirable for wound-healing dressing. At the low concentration of 1 mg/mL TiO_2_NPs used in this study, the antibacterial action was exerted only by the antibiotic, while the TiO_2_NPs had no effect. Overall, the Neomycin sulphate-loaded GelMA10%_1TiO_2_NP hydrogel exhibits key characteristics for tissue regeneration, such as mechanical strength, water balance, and antibacterial activity against *S. aureus*, making it a promising model for advanced wound-healing technologies. Our data suggest that GelMA10%_1TiO_2_NPs exhibit interesting functional characteristics for future applications in wound healing, involving drug loading or combinations of drugs that act synergistically, with the aim of reducing dosage and preventing drug resistance.

## Figures and Tables

**Table 1:** DLS characterization of TiO_2_NPs.

Concentration (mg/mL)	Z-Average (nm)	PDI	ζ Potential (mV)
0.5	3700 ± 72	0.619 ± 0.117	−24.9 ± 0.6
1.0	3800 ± 513	0.785 ± 0.147	−34.0 ± 0.8

**Table 2:** Disk diffusion test.

Samples	Zone of Inhibition (mm)
Neomycin sulphate (30 µg)	14 ± 1
GelMA10%	0 ± 0
GelMA10%–Neomycin sulphate	25 ± 0.5
GelMA10%_1TiO_2_NPs	0 ± 0
GelMA10%_1TiO_2_NPs–Neomycin sulphate	24 ± 1.5

## Data Availability

The original contributions presented in this study are included in the article/Supplementary Materials. Further inquiries can be directed at the corresponding authors.

## Supplementary Materials

The following supporting information can be downloaded at: https://www.mdpi.com/article/10.3390/nano16090536/s1, Table S1: BE (eV), FWHM (eV), atomic percentages (in the same signal) and proposed assignments for all measured signals related to the nanocomposite GelMA10% sample; Table S2: BE (eV), FWHM (eV), atomic percentages (in the same signal) and proposed assignments for all measured signals related to the Neomycin-loaded nanocomposite GelMA10% sample.
