# Supplementary material for: TiO2 Nanocomposite GelMA Film as Wound Dressing: Physicochemical, Structural, Mechanical Properties and Antibacterial Activity Against Staphylococcus aureus
Source: Nanomaterials (Basel). 2026 Apr 28;16(9):536. doi: 10.3390/nano16090536 (PMC13164887; doi:10.3390/nano16090536)
Supplement: Supplementary file 1 [file nanomaterials-16-00536-s001.zip › nanomaterials-4247040-supplementary.pdf]

TiO2 nanocomposite GelMA film as wound dressing: physico-chemical, structural, mechanical properties and antibacterial activity against Staphylococcus aureus

Barbara De Berardis*[1], Raffaella Pecci [1], Roberta Morlino [2], Pietro Ioppolo [1], Marco Ranaldi [3], Giovanna Iucci [3], Alessandro Ferrarini [3], Giuseppe D'Avenio [1], Giorgio De Angelis [1], Maria Grazia Ammendolia*[1].

1 National Centre Artificial Intelligence and Innovative Technologies for Health, Istituto Superiore di Sanità, 00161 Rome, Italy; raffaella.pecci@iss.it; pietro.ioppolo@iss.it; giuseppe.davenio@iss.it; giorgio.deangelis@iss.it;
2 Environment and Health Department, Istituto Superiore di Sanità, 00161 Rome, Italy; roberta.morlino@iss.it;
3 Department of Sciences, Roma Tre University, 00154 Rome, Italy; marco.ranaldi@uniroma3.it; giovanna.iucci@uniroma3.it; ale.ferrarini@ stud.uniroma3.it
*Correspondence: barbara.deberardis@iss.it; maria.ammendolia@iss.it

Table S1. BE (eV), FWHM (eV), atomic percentages (in the same signal) and proposed assignments for all measured signals related nanocomposite GelMA10% sample.

**Sample**		signal	BE (eV)	FWHM (eV)	Atomic %	Assignment
**GelMA**		C1s	285.0<br>286.2<br>287.5<br>289.1	1.56<br>1.56<br>1.56<br>1.56	66<br>24.1<br>7.7<br>2.3	C-C, C-H<br>C-N, C-O<br>N-C=O<br>O-C=O
		N1s	399.5<br>401.2	2.02<br>2.02	64.1<br>35.9	N-C=O<br>$N\text{-}H_3^+$
		$S2p_{3/2}$	162.4<br>164.5	2.2<br>2.2	39.7<br>60.4	C-S-C<br>C-(S=O)-C
		O1s	527.5<br>531.7<br>532.9	4.69<br>4.69<br>4.69	24.1<br>42.3<br>33.7	TiO_2 substrate<br>C=O<br>C-O

Table S2. BE (eV), FWHM (eV), atomic percentages (in the same signal) and proposed assignments for all measured signals related to the Neomycin-loaded nanocomposite GelMA10% sample.

**Sample**	signal	BE (eV)	FWHM (eV)	Atomic %	Assignment
**GelMA+Neo**	C1s	285.0<br>286.1<br>287.3<br>288.6	1.45<br>1.45<br>1.45<br>1.45	74.3<br>17.3<br>5.7<br>2.7	C-C, C-H<br>C-N, C-O<br>N-C=O<br>O-C=O

	N1s	399.3<br>401.3	2.56<br>2.56	60.2<br>39.8	N-C=O<br>$N-H_3^+$
	O1s	527.9<br>530.9<br>533.6	3.68<br>3.68<br>3.68	23.3<br>39.7<br>37.1	TiO_2 substrate<br>C=O<br>$C-O/H_2O$
	$S2p_{3/2}$	163.1<br>164.9	1.07<br>1.07	51.3<br>48.7	C-S-C<br>C-(S=O)-C
